# Exposure to the Gram-Negative Bacteria *Pseudomonas aeruginosa* Influences the Lung Dendritic Cell Population Signature by Interfering With CD103 Expression

**DOI:** 10.3389/fcimb.2021.617481

**Published:** 2021-07-06

**Authors:** Julyanne Brassard, Joanny Roy, Anne-Marie Lemay, Marie-Josée Beaulieu, Emilie Bernatchez, Marc Veillette, Caroline Duchaine, Marie-Renée Blanchet

**Affiliations:** Institut Universitaire de Cardiologie et de Pneumologie de Québec, Université Laval, QC, Canada

**Keywords:** dendritic cell (DC), *Pseudomonas aeruginosa*, Gram-negative bacteria, lung inflammation, lipopolysaccharide, CD103, granulocyte-macrophage colony-stimulating factor

## Abstract

Lung dendritic cells (DCs) are divided into two major populations, which include CD103^+^XCR1^+^ cDC1s and CD11b^+^Sirpα^+^ cDC2s. The maintenance of their relative proportions is dynamic and lung inflammation, such as caused by exposure to lipopolysaccharide (LPS), a component of the outer membrane of Gram-negative bacteria, can have a significant impact on the local cDC signature. Alterations in the lung cDC signature could modify the capacity of the immune system to respond to various pathogens. We consequently aimed to assess the impact of the Gram-negative bacteria *Pseudomonas aeruginosa* on lung cDC1 and cDC2 populations, and to identify the mechanisms leading to alterations in cDC populations. We observed that exposure to *P. aeruginosa* decreased the proportions of CD103^+^XCR1^+^ cDC1s, while increasing that of CD11b^+^ DCs. We identified two potential mechanisms involved in this modulation of lung cDC populations. First, we observed an increase in bone marrow pre-DC IRF4 expression suggesting a higher propensity of pre-DCs to differentiate towards the cDC2 lineage. This observation was combined with a reduced capacity of lung XCR1^+^ DC1s to express CD103. *In vitro*, we demonstrated that GM-CSF-induced CD103 expression on cDCs depends on GM-CSF receptor internalization and RUNX1 activity. Furthermore, we observed that cDCs stimulation with LPS or *P. aeruginosa* reduced the proportions of intracellular GM-CSF receptor and decreased RUNX1 mRNA expression. Altogether, these results suggest that alterations in GM-CSF receptor intracellular localization and RUNX1 signaling could be involved in the reduced CD103 expression on cDC1 in response to *P. aeruginosa*. To verify whether the capacity of cDCs to express CD103 following *P. aeruginosa* exposure impacts the immune response, WT and *Cd103^-/-^* mice were exposed to *P. aeruginosa*. Lack of CD103 expression led to an increase in the number of neutrophils in the airways, suggesting that lack of CD103 expression on cDC1s could favor the innate immune response to this bacterium.

## Introduction

Conventional dendritic cells (cDCs) play an important role in both innate and adaptive immune responses. In the lungs, cDCs are critical sentinel cells that capture, process and present antigens to activate naive T cells in lymph nodes. In addition, cDCs are involved in the innate immune response *via* cytokine and chemokine production (Macri et al., 2018). Lung conventional DCs comprise a variety of subsets that are typically subdivided into two sub-populations named cDC1s and cDC2s (Guilliams et al., 2014; Guilliams et al., 2016). cDC1s express the surface marker XCR1, and the expression of IRF8 and BATF3 transcription factors is required for their development. Additionally, they can express the alpha-E integrin CD103 in non-lymphoid organs like the lung, while they express CD8α in lymphoid tissues. cDC2s are characterized by IRF4, Sirpα and CD11b expression (Crozat et al., 2011; Guilliams et al., 2014; Gurka et al., 2015).

The majority of cDC development occurs in the bone marrow and requires the presence of the FMS-like tyrosine kinase 3 ligand (FLT3L) cytokine (Ginhoux et al., 2009). During this process, the commitment of cDC precursors to the cDC1 or cDC2 lineage happens relatively early in cDC development (Grajales-Reyes et al., 2015; Schlitzer et al., 2015). cDC precursors then leave the bone marrow at the pre-DC stage and migrate through the bloodstream to various organs such as the lung (Liu et al., 2009). Pre-DCs committed to the cDC1 lineage do not express CD103 (Brassard et al., 2019). The exact mechanisms by which cDC1s acquire CD103 expression upon their entrance in the lung remain unclear, but *in vivo* and *in vitro* studies suggest that exposure to GM-CSF, present in the lung, is a potent inducer of cDC CD103 expression (King et al., 2010; Greter et al., 2012; Mayer et al., 2014). To date, there is no information concerning transcription factors involved in cDC CD103 expression. However, the RUNX family of transcription factors is involved in the induction of CD103 expression in T cells (Grueter et al., 2005). The only reported ligand of αE integrin (CD103) is E-cadherin, which is expressed by epithelial cells (Corps et al., 2001). While the role of CD103 expression on cDC1s remains unclear, reports demonstrate that T cells CD103 expression facilitates lymphocyte localization and induces intracellular signaling (Pauls et al., 2001; Franciszkiewicz et al., 2013).

The two cDC subpopulations have distinct and often opposite functions (Schlitzer et al., 2015; Guilliams et al., 2016). cDC1s are particularly important for IL-12 production, antigen cross-presentation to CD8 T cells and CD4 T cell polarization into T_H_1 (Hildner et al., 2008; Mashayekhi et al., 2011; Martínez-López et al., 2015). The specific function of cDC2s is more controversial, but some studies suggested that they are important for T_H_2 polarization (Gao et al., 2013; Plantinga et al., 2013). To date, there is no consensus regarding the roles of cDC1s or cDC2s in the efficacy of antibacterial immune responses. Nevertheless, some interesting data suggest that, as observed in several other types of immune responses, cDC1s and cDC2s elicit distinctive functions in the fight against bacteria. Indeed, in most bacterial infections, polarization of naive T cells into T_H_1 improves bacterial clearance and lung function, which suggests a beneficial role for cDC1s (Moser et al., 1997; Moser et al., 2002). In accordance, in a mouse model of lung infection with the Gram-negative bacteria *Chlamydia muridarum*, CD103^+^ cDC1s induced a stronger T_H_1 polarization compared to cDC2s, and CD103^+^ cDC1 injection improved bacterial clearance (Shekhar et al., 2018). It should be noted, however, that cDC1s are usually involved in infections to intracellular bacteria, and that their role in response to *Pseudomonas aeruginosa* is not well-described. cDC2s, on the other hand, could be important for the innate immune response. Indeed, in response to LPS lung exposure, lung CD11b^+^ DCs produce more KC (CXCL1) and MIP–2 (CXCL2), two chemokines involved in neutrophil recruitment, compared to CD103^+^ cDC1s (Beaty et al., 2007).

These distinctive roles for cDC1s and cDC2s in the response to bacteria suggest that the tight balance between cDC lung subsets is important to support effective local immune responses. Recently, we demonstrated that lung exposure to lipopolysaccharide (LPS), which induces a strong local and peripheral inflammatory response, modulates the proportions of cDC populations by decreasing the percentage of CD103^+^ cDC1s and increasing CD11b^+^ DC proportions (Brassard et al., 2019). Since LPS is one of multiple bacterial components that may influence cDC populations and with the potential crucial role of cDC1s in the fight against bacterial infections in the lung, the impact of whole bacteria on the lung cDC signature remained an important unanswered question. We therefore set out to analyze the influence of an acute exposure to the Gram–negative bacteria *P. aeruginosa* on the local lung cDC signature. *P. aeruginosa* is ubiquitously found in nature and causes opportunistic acute and chronic infections in immunocompromised patients, such as those suffering of cystic fibrosis (Green et al., 1974; Lyczak et al., 2000; Moradali et al., 2017). We observed that *P. aeruginosa* modulated the proportions of lung cDC1 and cDC2 populations in favor of cDC2s, which was in part explained by a higher propensity of bone marrow cDC precursors to differentiate towards the cDC2 lineage, and by an incapacity of lung cDC1s to fully express CD103 in response to GM-CSF. The latter was linked to reduced GM–CSF receptor internal localization, following exposure to *P. aeruginosa* and LPS, and alterations in RUNX1 expression, which regulate CD103 expression. Finally, we report that the lack of CD103 expression on cDCs leads to an exacerbated airways neutrophilia, supporting the idea that the absence of CD103 expression on cDC1s promotes the lung innate response to *P. aeruginosa*. We therefore shed a light on a possible mechanism demonstrating that the blockade of cDC CD103 expression by Gram-negative bacteria is a crucial step in promoting the initial innate immune response to this potentially infectious agent in the lung.

## Material and Methods

### Mice

*Cd103^−/−^* (B6.129S2(C)-Itgae^tm1Cmp^/J) and wild-type (WT) mice were purchased from Jackson Laboratories and kept in a specific pathogen-free animal unit (Centre de recherche de l’Institut Universitaire de Cardiologie et de Pneumologie de Québec, Laval University, Québec, QC, Canada) for the duration of the experiments. *Cd103^−/−^* and WT mice were not co-housed during the duration of experiments. Experiments were approved by local ethics committees and followed Canadian animal care guidelines.

### Intranasal Instillation With *P. aeruginosa* and LPS

Non-mucoid *P. aeruginosa*, strain Boston 41501 (ATCC #27853, Manassas, VA, USA) was incubated overnight in tryptic soy broth (TSB) (Wisent, St-Bruno, QC, CA) at 37°C in a rotating shaker and 1 ml of the suspension was re-incubated in new TSB media for 2 h. Bacteria were washed and diluted in saline, and the desired concentration was adjusted by spectrophotometry according to a reference curve. Bacterial concentration was systematically verified by quantitative culture of the inoculum. Age- and sex-matched WT and *Cd103^-/-^* mice received a 50 μL intranasal (i.n.) instillation of 5 x 10^5^ or 5 x 10^6^ colony forming units (CFU) of *P. aeruginosa* or 350 ng of LPS (Sigma-Aldrich, St. Louis, MO USA). Mice were euthanized at 2, 6 or 18 h following LPS or *P. aeruginosa* exposure. Bronchoalveolar lavages (BAL) were obtained *via* three injections/aspirations of 1 mL of saline, in mice euthanized at 2 or 6 h post i.n. Total BAL cells of LPS-treated mice were counted and differential counts were determined on Giemsa stained cytospins (HemaStain Set, Thermo Fisher Scientific, Waltham, MI, USA). The BAL composition of *P. aeruginosa*-exposed mice was analyzed by flow cytometry and neutrophils were identified as auto-fluorescence^-^, CD45^+^, Ly-6G^+^ and CD11b^+^ and macrophages were identified as auto-fluorescence^+^, CD45^+^, CD11c^+^ and Siglec-F^+^. For flow cytometry analysis, the lung, spleen, femur and tibia were collected in phosphate buffered saline (PBS) 18 h after lung i.n. instillation with *P. aeruginosa*.

### Leukocyte Isolation

Lung leukocytes were obtained by the digestion of lung tissue with 200 U/ml collagenase IV (Sigma-Aldrich) for 45 min at 37°C. Digested lungs and spleens were pressed through a 70-μm cell strainer. Bone marrow cells were isolated by flushing the cells from tibias and femurs using a 25 Gauge needle with PBS. Red blood cells were lysed with ammonium chloride and cDC or cDC precursors were analyzed by flow cytometry.

### FLT3L-BMDCs

Bone marrow cells were isolated as described in the leukocyte isolation section. Cells were cultured at 1.5 x 10^6^ cells/ml for 7 days in RPMI 1640 media (Wisent) supplemented with 10% FBS (Wisent), 50 µM β-mercaptoethanol, antibiotic-antimycotic (Wisent) and 100 ng/ml FMS-like tyrosine kinase 3 ligand (FLT3L) (peprotech, Rocky Hill, NJ, USA, catalog no. 250-31L). On day 7, BMDCs were harvested for stimulation.

### Spleen-Isolated cDCs

To expand cDC populations *in vivo*, WT mice were subcutaneously injected in the lower back with 5 x 10^5^ FLT3L-producing B16 melanoma cells, previously grown in DMEM media (Wisent) supplemented with 10% FBS. When the tumor reached 1 cm diameter, mice were euthanized and the spleen collected. Spleen leukocytes were isolated as described in leukocyte isolation section. cDCs were purified by negative selection using the EasySep Mouse pan-DC Enrichment Kit (StemCell Technologies, Vancouver, BC, Canada).

### DCs *In Vitro* Stimulation

10^6^ cells/ml of splenic or FLT3L-BMDCs were stimulated with 10 ng/ml Granulocyte-macrophage colony-stimulating factor (GM-CSF) (Peprotech, catalog no. 315-03), 10 ng/ml LPS (Sigma-Aldrich) or *P. aeruginosa* at a ratio of 1 cDC: 1 P*. aeruginosa* for spleen-isolated cDC or 10 DCs: 1 P*. aeruginosa* for FLT3L-BMDCs in RPMI 1640 supplemented with 10% FBS and 50 µM β-mercaptoethanol for 12, 18 or 48 h. For some experiments, FLT3L-BMDCs were pre-treated with 40, 80 or 120 μM of the dynamin inhibitor Dynasore (Sigma-Aldrich) or with 10, 25 or 50 μM of CBFβ-Runx1 Inhibitor II (Sigma-Aldrich). Following stimulation, CD103, GM-CSFRα and RUNX1 protein or mRNA expression were analyzed by flow cytometry and qRT-PCR respectively.

### Flow Cytometry

BAL leukocytes, tissue-isolated leukocytes or *in vitro*-stimulated cDCs were stained with TruStain FcX anti-mouse CD16/32 antibody (BioLegend, San Diego, CA, USA) and CD45-APC-Cy7, CD103-PE, CD11b-PeCy7, CD11c-BV711, I-A/I-E (MHC II)-Pacific Blue, I-A/I-E (MHC II)-PERCP, CD172a (Sirpα)-APC-Cy7, CD19-biotin, CD90.2-biotin, IRF4-PE, Ly-6G-PE, XCR1-APC, CD8α-APC-Cy7, Lineage antibody cocktail-Pacific Blue, CD135 (FLT3)-biotin, Streptavidin-PERCP (BioLegend), NK1.1-biotin (ablab, Vancouver, BC, CA), CD11c-APC, Siglec-F-BV711 (BD Biosciences, San Jose, USA), IRF8-APC (Miltenyi Biotec, Bergisch Gladbach, Allemagne) and Streptavidin-Pe-Cy7 (eBioscience, Thermo Fisher Scientific), GM–CSFRα-APC (R&D system, Minneapolis, MN, USA). Total, neutrophils and macrophages BAL number were determined using precision count beads (BioLegend). Intracellular staining was performed using the True-Nuclear™ Transcription Factor Buffer Set (BioLegend) according to the manufacturer’s instructions. Cells were analyzed using a BD LSR Fortessa cytometer (BD Biosciences) and FlowJo software V10 (BD, Franklin Lakes, NJ, USA). Mean fluorescence intensity (MFI) data were analyzed as Δ MFI, which corresponds to the MFI of the antigen-positive population minus the MFI of the fluorescence minus one (FMO) control of this population.

### Real-Time PCR Analysis

1.5 x 10^6^ FLT3L-BMDCs were stimulated with GM-CSF, LPS or *P. aeruginosa* for 12 h and RNA was isolated using RNAspin Mini Kit (GE Healthcare Life Sciences, Chicago, USA) and reverse transcribed with an iScript cDNA Synthesis Kit (Bio-rad, Mississauga, Ontario, CA). Real-time PCR analysis was performed for CD103 (Itgae), GM-CSFRα (Csf2ra) and RUNX1 using the Rotor-Gene 6000TM (Qiagen, Valencia, CA, USA) in Sso Advanced Universal SYBR Green Supermix (Bio-rad). The following primers (IDT, Coralville, USA) were used: Itgae (forward: 5’-AGGTCATAGATACGGTCAGGT-3’, reverse: 5’-GGTTAGATTTCAATGGCGATGG-3’), GM-CSFRα (forward: 5’-CCTCACCATCCATCGCA-3’, reverse: 5’-GAAGCAGTAGCGTGGAGAAG -3’), RUNX1 (forward: 5’-GTAGCGAGATTCAACGACCTC-3’, reverse: 5’-TCTATGGTAGGTGGCAACTTG-3’). Expression was normalized to the Gnb and Rplp0 mRNA expression validated for stability of expression in this model.

### Enumeration of Colony Forming Unit in BAL and Lung

Lungs were homogenized in 1 ml of saline using the Polytron Tissue Homogenizer (Kinematica, Luzern, Switzerland). Homogenates and BALs were subjected to 10-fold serial dilutions in saline and cultured in tryptic soy agar (Wisent) at 37°C and CFUs were counted 18 to 24 h later.

### Statistics

Data are presented as mean ± SEM. Graphpad Prism version 8 (San Diego, CA, USA) was used to analyze all data. Statistical analysis for multiple comparisons was performed using an ANOVA table followed by Tukey’s multiple comparison tests. Non-multiple comparisons were analyzed using paired or unpaired T-tests. Statistical significance was determined at p < 0.05.

## Results

### Lung Exposure to the Gram-Negative Bacteria *Pseudomonas aeruginosa* Leads to a Major Modulation of DC Populations

**Figure 1:**
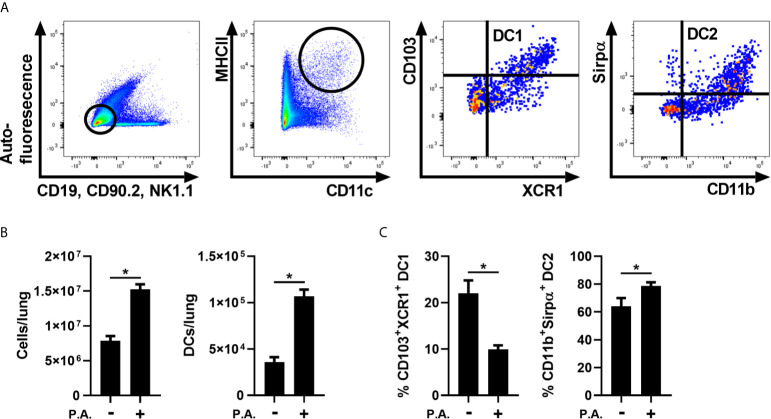
Lung exposure to *P. aeruginosa* altered the proportions of cDC1s and cDC2s in favor of cDC2s. WT mice were exposed to a single i.n. instillation of 5 x 10^5^ CFU of *P. aeruginosa* (P.A.) and mice were euthanized 18 h later. Lung cDC populations were analyzed by flow cytometry. **(A)** Sequential gating strategy used to identify total cDCs (auto-fluorescence^-^, CD19^-^, CD90.2^-^, NK1.1^-^, MHC II^Hi^ and CD11c^+^), cDC1 (CD103^+^XCR1^+^) and cDC2 (CD11b^+^Sirpα^+^). **(B)** Number of total lung cells and cDCs **(C)** Percentage of CD103^+^XCR1^+^ cDC1 and CD11b^+^Sirpα^+^ cDC2 in lung cDCs. Data are presented as mean ± SEM; n = 8-9 mice per group combined from two separate experiments *p < 0.05 using unpaired t-test.

We first aimed to verify whether lung exposure to whole Gram-negative bacteria influences the CD103^+^ cDC1 and cDC2 population ratios in the lung. To test this, lung cDC populations were analyzed 18 h following i.n. exposure with *P. aeruginosa*. Lung cDCs were identified as auto-fluorescence^-^, CD19^-^, CD90.2^-^, NK1.1^-^, MHC II^Hi^ and CD11c^+^. CD103^+^ cDC1 characterization was based on CD103 and XCR1 expression, whereas CD11b and Sirpα markers were used to identify cDC2s (**Figure 1A** and **Supplementary Figure 1** for full gating strategy). We first observed that *P. aeruginosa* induced an important increase in lung total cells, which was accompanied by a two-fold increase in cDC numbers (**Figure 1B**). We also report a decrease in CD103^+^XCR1^+^ cDC1 proportions and, in return, an increase in CD11b^+^Sirpα^+^ cDC2 proportions (**Figure 1C**). These results indicate that, as observed previously with LPS (Brassard et al., 2019), *P. aeruginosa* modulates lung cDC populations in favor of the DC2/monocyte-derived DC population.

### Lung Exposure to *Pseudomonas aeruginosa* Influences Bone Marrow cDC Precursors

**Figure 2:**
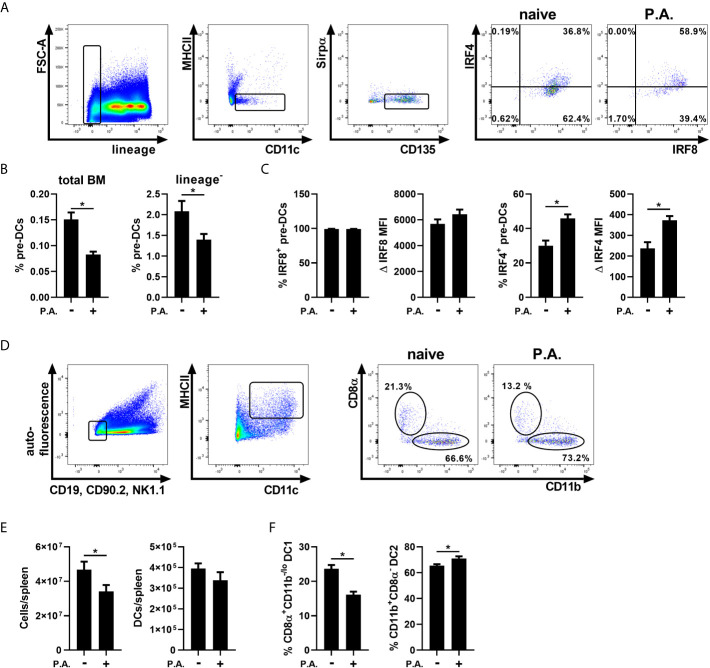
Intranasal instillation with *P. aeruginosa* induced a systemic effect on bone marrow cDC precursors and spleen cDC populations. WT mice were euthanized 18 h following i.n. instillation of 5 x 10^5^ CFU of *P. aeruginosa* (P.A.) and bone marrow and spleen cells were analyzed by flow cytometry. **(A)** Bone marrow pre-DCs sequential gating strategy (lineage^-^, MHC II^-^, CD11c^+^, Sirpα^lo/-^, CD135^+^) and representative flow cytometry profile of IRF4 and IRF8 expression in pre-DCs from naive and *P. aeruginosa* treated mice. **(B)** Percentage of bone marrow pre-DCs in total bone marrow cells and lineage^-^ cells. **(C)** Bone marrow pre-DCs: percentage and Δ MFI of IRF8 and IRF4. **(D)** Spleen cDCs sequential gating strategy (auto-fluorescence^-^, CD19^-^, CD90.2^-^, NK1.1^-^, MHC II^Hi^ and CD11c^+^) and representative flow cytometry profile of CD8α and CD11b expression on cDCs from naive and *P. aeruginosa* treated mice. **(E)** Total spleen cells and spleen cDC number. **(F)** Percentage of CD8α^+^CD11b^-^ cDC1 and CD11b^+^CD8α^-^ cDC2 from spleen total cDCs. Data are presented as mean ± SEM; n = 8-10 mice per group combined from two separate experiments except for panel **(C)**, IRF4; n = 5 representative of three separate experiments. *p < 0.05 using unpaired t-test.

The commitment to the cDC1 or cDC2 lineage is defined before the pre-DCs stage, and can be influenced by peripheral inflammation (Schlitzer et al., 2015; Grajales-Reyes et al., 2015; Meyer et al., 2018; Beshara et al., 2018; Brassard et al., 2019). Thus, we hypothesized that following i.n. instillation with *P. aeruginosa*, a shift towards the cDC2 fate could support the accumulation of cDC2s in the lung. cDC1 and cDC2-committed precursors both express IRF8 initially, but the further commitment to the cDC2 lineage results in a decrease in IRF8 expression in time. In contrast, only cDC2-committed precursors express IRF4 at the later stage of development (Sichien et al., 2016). To determine whether lung exposure to *P. aeruginosa* alters the cDC1 vs cDC2 commitment, the expression of these two transcription factors was analyzed in bone marrow pre-DCs (Guilliams et al., 2014; Grajales-Reyes et al., 2015). Pre-DCs were identified as lineage^-^, MHC II^-^, CD11c^+^, Sirpα^-/lo^, CD135 (FLT3)^+^ (**Figure 2A** and **Supplementary Figure 2**) (Schlitzer et al., 2015; Grajales-Reyes et al., 2015). We report that the percentage of pre–DCs from total bone marrow cells and lineage^-^ cells was significantly decreased in mice exposed to *P. aeruginosa* (**Figure 2B**). However, this was accompanied by an increase in IRF4 expression following lung instillation with *P. aeruginosa* (**Figure 2C**). This result suggests a propensity of pre-DCs to differentiate towards the cDC2 lineage and a strong exodus of pre-DCs in response to *P. aeruginosa*.

To verify whether this impact was lung-specific, we tested cDC subsets in tissues that are not in direct contact with *P. aeruginosa*, such as the spleen. Total splenic cDCs were identified with the same gating strategy used in the lung, but, as spleen is a lymphoid organ, the CD8α surface marker was evaluated on cDC1s (Edelson et al., 2010; Guilliams et al., 2014). Thus, CD8α^+^CD11b^-^ cDCs were characterized as cDC1s, while CD11b^+^CD8α^-^ cDCs were classified as cDC2s (**Figure 2D**, **Supplementary Figure 3**) (Guilliams et al., 2014; Tavernier et al., 2015; Guilliams et al., 2016). Total splenic cells were decreased following i.n. instillation with *P. aeruginosa*, while cDC number remained unchanged (**Figure 2E**). The splenic proportion of CD8α^+^CD11b^-^ cDC1s was decreased and in contrast the CD11b^+^CD8α^-^ cDC2 proportion was increased, suggesting a systemic impact of *P. aeruginosa* on cDC signatures in various tissues (**Figure 2F**).

### *Pseudomonas aeruginosa* Interferes With the Capacity of cDCs to Express CD103

**Figure 3:**
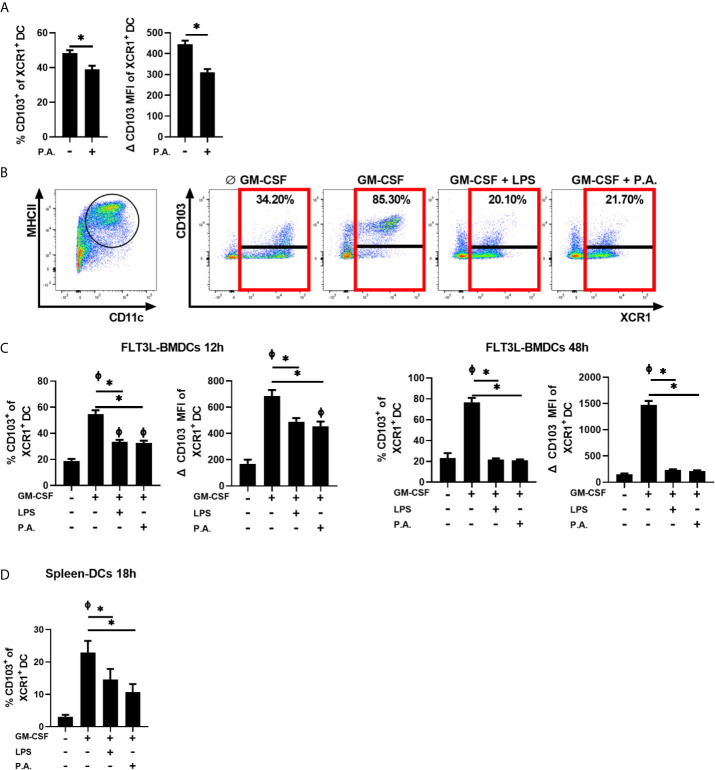
*P. aeruginosa* stimulation prevents GM-CSF-induced CD103 expression on cDCs. **(A)** Mice were euthanized 18 h after a single i.n. instillation of 5 x 10^5^ CFU of *P. aeruginosa* (P.A.) and the percentage of CD103^+^ cells and CD103 MFI in XCR1^+^ cDC1 gated lung cells were analyzed by flow cytometry. **(B, C)** FLT3L-BMDCs or **(D)** spleen-isolated cDCs were stimulated with GM-CSF ± LPS or *P. aeruginosa* (P.A.) for 12 h, 18 h or 48 h and CD103 and XCR1 expression were analyzed by flow cytometry. **(B)** Gating strategy used to identify MHC II^Hi^CD11c^+^ cDCs and representative flow cytometry profile of CD103 expression on XCR1^+^ FLT3L-BMDCs stimulated for 48 h. **(C, D)** Percentage of CD103^+^ cells and CD103 MFI from previously gated XCR1^+^ cDCs. Data are presented as mean ± SEM; (**A** % CD103) n = 10-11 pooled from two separate experiments, (**A** CD103 MFI) n = 5 representative of three separate experiments. (**C, D** % CD103) n = 5-6 samples of cDCs per group pooled from two separate experiments. (**C, D** CD103 MFI) n = 3 representative of two separate experiments. * = p < 0.05 compared between conditions stimulated with GM-CSF. Φ = p < 0.05 compared to unstimulated condition. P-values were analyzed using unpaired t-test **(A)** or paired-one-way ANOVA **(C, D)**.

We previously demonstrated that LPS and inflammatory factors directly abrogate the induction of CD103 expression on cDC1s (Brassard et al., 2019). To verify whether exposure to whole bacteria also influences CD103 expression on cDC1s, the proportion of XCR1^+^ cDC1s expressing CD103 was analyzed 18 h following i.n. instillation with *P. aeruginosa*. We observed a significant decrease in the percentage of CD103^+^ cDCs within the cDC1 population in response to bacterial exposure (**Figure 3A**).

Furthermore, we verified whether *P. aeruginosa* directly interferes with the capacity of cDC1s to express CD103 in response to GM-CSF using FLT3L-derived bone marrow DCs (BMDCs) (Gating strategy, **Figure 3B** and **Supplementary Figure 4**). Without any stimulation, the percentage of XCR1^+^ BMDCs expressing CD103 was low, with a mean of 18% positive cells. As expected, a 12 h stimulation with GM-CSF increased CD103 expression on cDC1s to approximately 55% (**Figure 3C**). However, the presence of *P. aeruginosa* during the GM-CSF stimulation prevented the maximal induction of CD103 (**Figure 3C**) to a level similar to that of LPS. This incapacity of XCR1^+^ BMDCs to fully express CD103 worsened in time, as CD103 expression on XCR1^+^ cDCs was almost entirely abrogated in response to LPS and *P. aeruginosa* at 48 h (**Figures 3B, C**). Although few freshly isolated splenic cDC1s express CD103, its expression can be induced on splenic cDCs by GM-CSF stimulation (Sathe et al., 2011; Brassard et al., 2019). We report that the induction of CD103 expression by GM-CSF is also reduced on spleen–isolated cDCs following exposure to *P. aeruginosa*, and to a level that is similar to LPS exposure (**Figure 3D**). Therefore, the blockade of CD103 expression on cDCs by these stimuli seems to be independent of the method used to generate cDCs. Of note, neither LPS nor *P. aeruginosa* altered cDCs viability (data not shown). These results also suggested that the presence of *P. aeruginosa* in the lung can directly influence the capacity of newly-recruited cDC1s to express CD103 in response to local GM–CSF.

### *Pseudomonas aeruginosa* Influences the Localization of the GM-CSF Receptor

**Figure 4:**
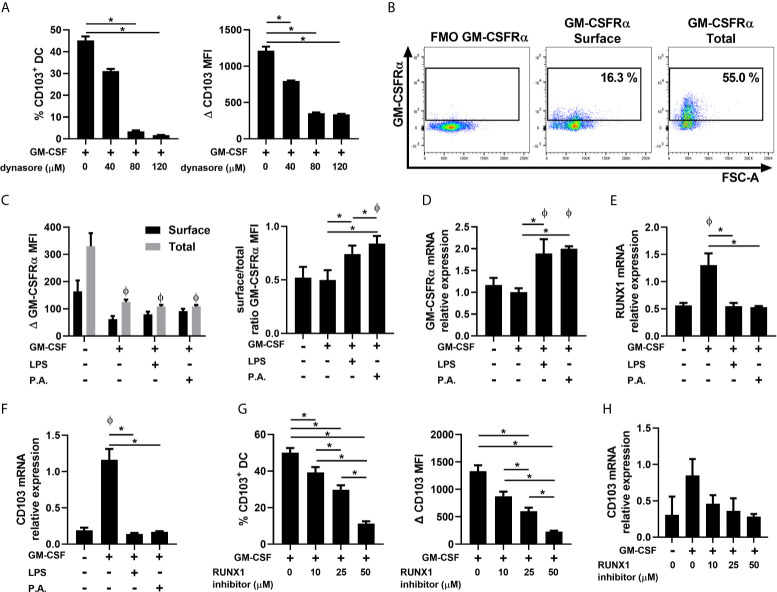
*P. aeruginosa* influences GM-CSFR localization on BMDCs. FLT3L–BMDCs were stimulated for **(A–G)** 48 h or **(B–F, H)** 12 h with GM-CSF ± **(A)** Dynasore, **(B–F)** GM-CSF ± LPS or *P. aeruginosa* (P.A.) or **(G, H)** a RUNX1 inhibitor. **(A–G)** Percentage of CD103^+^ and Δ CD103 MFI on cDCs. **(B)** Representative flow cytometry profile of surface and total GM–CSFRα expression on FLT3L-BMDCs. **(C)** Surface (extracellular) and total (extracellular + intracellular) Δ MFI (first panel) of GM-CSFRα, and ratio of surface to total expression of GM-CSFRα (second panel). **(D)** mRNA relative expression of GM-CSFRα (*Csf2ra*). **(E)** mRNA relative expression of RUNX1. **(F, H)** mRNA relative expression of CD103 (*Itgae*). Data are presented as mean ± SEM; **(A)** n = 3 representative of two separate experiments. **(C–H)** n = 4-6 samples of cDCs per group pooled from two separate experiments. *p < 0.05 compared between conditions stimulated with GM–CSF. Φ = p < 0.05 compared to unstimulated condition. P-values were analyzed using paired-one-way and two-way ANOVA.

The exact mechanisms by which GM-CSF induces CD103 expression remain unknown, but binding of GM-CSF to its receptor (GM-CSFR) leads to a signaling cascade that is mediated in part by GM-CSFR internalization (Broughton et al., 2012; Zsiros et al., 2019). To first test whether GM-CSFR internalization is indeed involved in GM-CSF-induced cDC CD103 expression, FLT3L–BMDCs were pre-treated with Dynasore, a dynamin inhibitor that blocks internalization of receptor–ligand complex, prior to GM-CSF stimulations (Macia et al., 2006; Zsiros et al., 2019). Pre-treatment with Dynasore blocked CD103 expression on FLT3L-BMDCs suggesting that GM-CSFR internalization is required for its expression on cDCs (**Figure 4A**).

To better understand whether GM-CSFR internalization is impacted by LPS or *P. aeruginosa*, extracellular and intracellular protein levels of the GM-CSFRα subunit were analyzed (**Figures 4B, C**). The combination of LPS or *P. aeruginosa* with GM-CSF did not alter total GM-CSFR expression (**Figure 4C**). However, when they were combined with GM-CSF, a higher ratio of surface to total GM-CSFRα expression was noted, indicating a reduced intracellular localization of the GM–CSFRα subunit (**Figure 4C**). This was not due to alterations in new receptor synthesis, as *csf2ra* (GM–CSFRα) mRNA expression was significantly increased in BMDCs exposed to *P. aeruginosa* (**Figure 4D**).

Additionally, RUNX1 mRNA expression, a member of the RUNX transcription factor family, was increased following GM-CSF stimulation, and abrogated in the presence of LPS or *P. aeruginosa* (**Figure 4E**). Following the same pattern as RUNX1, CD103 mRNA synthesis was also altered in the presence of LPS or *P. aeruginosa* (**Figure 4F**). To confirm the involvement of RUNX1 in the induction of CD103 expression in cDCs, FLT3L-BMDCs were pre-treated with a RUNX1 inhibitor prior to GM-CSF stimulation. We observed that the suppression of the transactivation activity of RUNX1 and its cofactor CBF in BMDCs leads to a reduction in both CD103 protein and mRNA synthesis, linking RUNX1 to CD103 expression in cDCs (**Figures 4G, H**). Therefore, our results suggest that the presence of *P. aeruginosa* impacts CD103 expression on cDC1s by interfering with the intracellular localization of the GM-CSFR, and by preventing RUNX1 mRNA expression.

### Lack of CD103 Expression Enhanced the Lung Innate Immune Response to *Pseudomonas aeruginosa*

**Figure 5:**
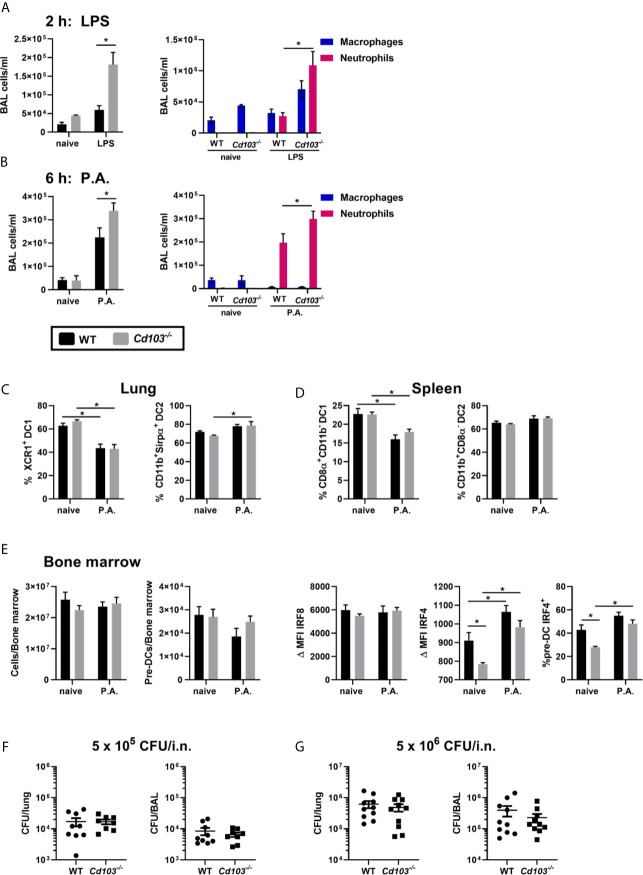
Lack of CD103 expression favors the recruitment of bronchoalveolar neutrophils. **(A, B)** Total number (first panel), macrophages and neutrophils (second panel) of bronchoalveolar lavage (BAL) cells were compared between WT and *Cd103^-/-^* mice **(A)** 2 h following LPS i.n. instillation or **(B)** 6 h following i.n. instillation of 5 x 10^5^ CFU of *P. aeruginosa* (P.A.). **(C–E)** Lung, spleen and bone marrow cells were analyzed by flow cytometry 18 h after i.n. instillation with 5 x 10^5^ CFU of *P. aeruginosa.* **(C)** Percentage of XCR1^+^ cDC1s and percentage of CD11b^+^Sirpα^+^ cDC2s in lung. **(D)** Percentage of CD8α^+^CD11b^+^ cDC1s and percentage of CD11b^+^CD8α^-^ cDC2s in spleen. **(E)** Total cell number, pre-DC number, ΔIRF8 MFI, ΔIRF4 MFI and percentage of IRF4^+^ pre-DCs in bone marrow. **(F, G)** *P. aeruginosa* CFU number present in the lung and BAL 6 h after i.n. instillation of **(F)** 5 x 10^5^ CFU or **(G)** 5 x 10^6^ CFU of *P. aeruginosa.* Data are presented as mean ± SEM; **(A)** n = 2-5 (naive-P.A.) representative of two independent experiments. **(B)** n = 3-10 (naive-P.A.) pooled from two separate experiments. **(C–E)** (total and pre-DC number) **(F, G)** n = 9-10 pooled from two separate experiments. **(E)** (IRF4-IRF8) n = 5 representative of two independent experiments. *p < 0.05 using two-way ANOVA.

Lung exposure to *P. aeruginosa* resulted in an increase in XCR1^+^ cDC1s that lack CD103 expression. To determine the impact of an incapacity to express CD103 by cDCs on the immune response, we sought to verify whether the absence of CD103 expression on cDC1s modulates the early innate immune response to LPS and *P. aeruginosa*. LPS or *P.* *aeruginosa* were injected intranasally into WT and *Cd103^-/-^* mice and cells from the bronchoalveolar lavage (BAL) were analyzed 2 h (LPS) and 6 h (*P. aeruginosa*) later. These times were chosen since CD103 is still expressed on cDCs at that time following LPS exposure (Brassard et al., 2019), and because the increase in total cells in response to *P. aeruginosa* is slower than following LPS administration (data not shown). Of note, although CD103 is also present on T cells, they do not play a role in the rapid innate immune response to these agents (Andrew et al., 1996; Bernatchez et al., 2017). Following the i.n. administration of LPS, total cells and neutrophil numbers were increased in the BAL of *Cd103^-/-^* mice compared to WT mice (**Figure 5A**). This was also observed in *Cd103^-/-^* mice exposed to *P. aeruginosa* compared to WT (**Figure 5B**). These results suggest that the absence of CD103 expression facilitates neutrophil recruitment in response to Gram–negative bacteria.

We then assessed whether this was caused by a modulation in cDC or cDC precursors populations. The lung and spleen total cell numbers, cDC numbers, (data not shown) and the percentage of cDC1s and cDC2s were similar between WT and *Cd103^-/-^* mice in naive and *P. aeruginosa* exposed mice (**Figures 5C, D**). Moreover, bone marrow total cells, pre–DC numbers and IRF8 MFI were also similar between strains (**Figure 5E**). The IRF4 MFI and the percentage of IRF4+ pre-DCs were significantly lower in *Cd103^-/-^* naive mice compared to WT naive mice, but similar in both strains following the exposure to *P. aeruginosa* (**Figure 5E**). Therefore, the higher recruitment of lung neutrophils in *Cd103^-/-^* mice is not caused by a difference in number or proportions in cDC or pre-DC populations.

To determine whether this affected bacterial clearance, we first studied the optimal time to study *P. aeruginosa* clearance. Almost all bacteria were cleared at 12 h following i.n. instillation of *P. aeruginosa* (data not shown), thus the 6 h timepoint was selected to compare CFU number in WT and *Cd103^-/-^* mice. We report similar numbers of *P. aeruginosa* CFU counts in the BAL and lung homogenates of the two mouse strains, (**Figure 5F**) and this was independent of the concentration used (**Figure 5G**). This likely indicates that the bacterial clearance kinetics of this model/*P. aeruginosa* strain may be too quick to verify whether the increase in neutrophils observed in the absence of cDC CD103 expression leads to better bacterial clearance.

## Discussion

**Figure 6:**
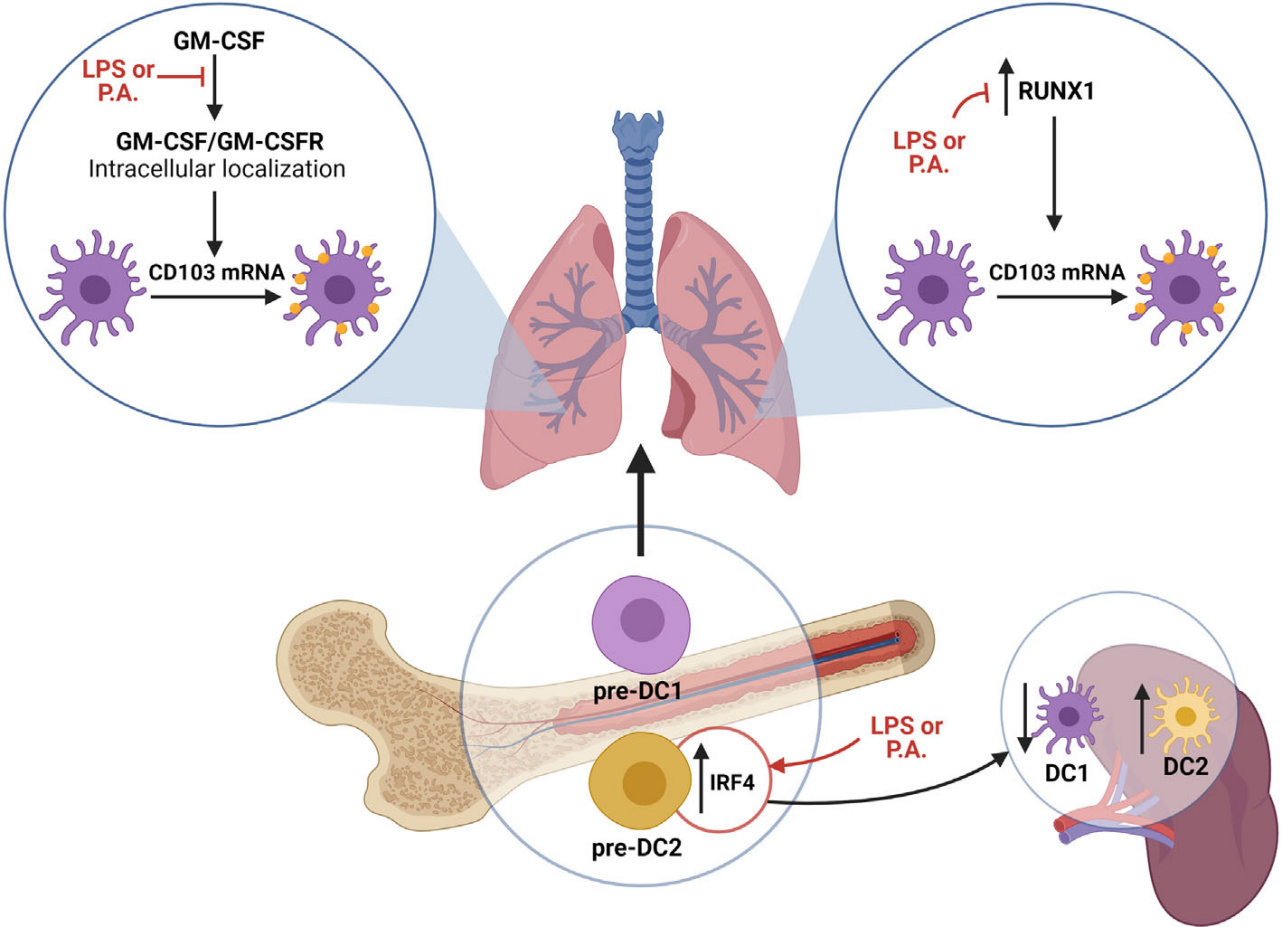
Schematic representation of proposed mechanisms supporting the modulation of lung cDC populations in response to *P. aeruginosa.* LPS and *P.* *aeruginosa* directly impact the capacity of cDCs to internally localize the GM-CSFR, which is a crucial step in GM-CSF signaling leading to CD103 mRNA synthesis and CD103 expression on cDC1s. Additionally, LPS and *P. aeruginosa* interfere with RUNX1 expression, whose activity is needed for GM-CSF-dependent CD103 expression. Finally, exposure to LPS and *P. aeruginosa* alters the pre-DC transcriptional signature, favoring cDC2 precursor differentiation. Altogether, the exposure to LPS and *P. aeruginosa* impacts the capacity of cDCs to express CD103, as well as the cDC precursor signature. **Figure 6** was Created with BioRender.com.

DCs take part in the induction of innate and adaptive immune responses, and the efficacy of these responses is influenced by the nature of local cDC subsets (Macri et al., 2018). Until now, few studies focused on the influence of bacterial infection on cDC populations. In this report, we demonstrated that lung exposure to live *P. aeruginosa* bacteria decreases the proportions of CD103^+^ cDC1s in favor of CD11b^+^ cDC2s/monocyte-derived DCs. We determined that this was in part modulated by modifications in bone marrow pre-DC populations and an altered CD103 expression on XCR1^+^ cDC1s (**Figure 6**). Furthermore, we demonstrated that the absence of CD103 expression increases neutrophils recruitment in the lung in response to LPS and *P. aeruginosa*.

First, we realize that our gating strategy to identify cDC2s does not discard monocyte-derived DCs, so this population must be considered as potentially upregulated in response to *P. aeruginosa*. Independently of this, the altered proportions of cDC populations are not restricted to lung exposure to LPS or *P. aeruginosa*. Indeed, similar results were reported in a mouse model of lung infection with *Chlamydia muridarum*, an LPS^+^ bacteria (Shekhar et al., 2018; Brassard et al., 2019). Interestingly, in the *C. muridarum* model, a decrease in CD103^+^ cDC proportions was observed up to 7 days post-infection, suggesting that this phenomenon is not limited to acute immune responses (Shekhar et al., 2018). This is also supported by similar alterations observed by our group in a 21-day mouse model of chronic allergic inflammation to *Saccharopolyspora rectivirgula* antigen (Bernatchez et al., 2017).

Our results suggest that lung exposure to *P. aeruginosa* altered bone marrow cDC precursors towards pre-DCs that are committed to the cDC2 lineage, which likely contributes to the accumulation of lung cDC2s. A modification of bone marrow pre-DCs towards the cDC1 or cDC2 lineage was observed in other immunological contexts including cancer, viral infections and lung exposure to LPS (Beshara et al., 2018; Meyer et al., 2018 Brassard et al., 2019), suggesting that this mechanism is conserved across various diseases and types of inflammatory responses. We previously observed that lung exposure to LPS leads to a decrease in IRF8 expression in pre-DCs, whereas an increase of IRF4 expression is reported here in response to *P. aeruginosa* (Brassard et al., 2019). Some studies suggest a sequential order between IRF8 and IRF4 expression in which the decrease in IRF8 expression precedes the increase in IRF4 expression in pre-DCs committed to the cDC2 lineage (Bajaña et al., 2016; Sichien et al., 2016). Therefore, the differences reported in both studies could be explained by a difference in the timing of induction for these transcription factors in these models. Despite this difference, both results report an imbalance towards the cDC2 lineage differentiation.

Our results also suggest that splenic cDC populations are affected by lung *P. aeruginosa* exposure. As the cDC turnover in the spleen is fast, even at steady state, an absence of total cDC number increase (such as observed here) does not indicate an absence of new cDC recruitment (Kamath et al., 2002), and the altered cDC populations is likely a reflection of a systemic impact of *P. aeruginosa*. Thus, our results suggest that bone marrow pre-DCs are biased towards the cDC2 lineage, which in turns influences the subsets of cDC precursors recruited in the spleen, resulting in a modulation of splenic cDC1 and cDC2 proportions (**Figure 6**). As pre-DCs are precursors for most tissue cDCs, this modification in bone marrow pre–DCs could also influence cDC populations in other organs, and ultimately impact the efficacy of immune responses in case of a systemic infection (Ginhoux et al., 2009). We are aware that other mechanisms besides alterations in cDC precursors could be involved in explaining the differences in the cDC population signature in our models. As our gating strategy also included mo-DCs as CD11b^+^, this population could contribute to the imbalance reported here (Guilliams et al., 2016). Also, a higher migratory rate of cDC1s from the lung to the draining lymph nodes could take part in the observed decrease percentage of this population following *P. aeruginosa* exposure (Ho et al., 2011; Nakano et al., 2013).

Supported by previously-published results (Brassard et al., 2019) and present data, we propose that under homeostatic conditions, newly-recruited lung pre-DCs acquire CD103 expression in response to GM-CSF stimulation through their final differentiation into cDCs. However, in the context of Gram-negative bacterial exposure, the interaction with the bacteria or LPS prevents CD103 mRNA synthesis resulting in lower GM-CSF-induced CD103 expression on cDC1s. Importantly, our results do not demonstrate that LPS nor *P. aeruginosa* reduce the expression of CD103 once it has been expressed, but rather block the induction upon contact with GM-CSF. Furthermore, our observations suggest that a reduced internal localization of the GM-CSFR could be one of the mechanisms contributing to the reduction in CD103 expression. Our results linking the GM-CSFR internalization to CD103 expression were obtained using Dynasore a GTPase inhibitor that inhibits dynamin activity, which prevents endocytosis. Although this inhibitor is frequently used to block GM-CSFR internalization (Katz et al., 2016; Zsiros et al., 2019), Dynasore could also exert other effects. For instance, the presence of Dynasore could also block the internalization of other receptors, such as TLR4, express by cDCs (Kagan et al., 2008). However, as our assays with Dynasore were performed using only GM-CSF stimulation, it is very likely that in this case, the inhibition of CD103 expression was linked to a reduced blockade of GM-CSFR internalization. The exact steps linking the activation of GM-CSFR signaling to the induction of CD103 mRNA expression on cDCs remained unknown. For the first time, we demonstrate that RUNX1 expression is crucial for CD103 expression in cDCs. Additionally, we show that LPS and *P. aeruginosa* alter RUNX1 expression, likely contributing to reduced CD103 expression in response to these stimuli. Of course, other cellular signaling pathways besides GM-CSF are involved in regulating CD103 expression on cDCs. For example, TGFβ and retinoic acid also reportedly induced CD103 expression (Iliev et al., 2009; Roe et al., 2017; Roe et al., 2020). Whether LPS and *P. aeruginosa* alter CD103 expression in response to these stimuli remains unknown, but definitely of interest.

Despite a wealth of knowledge on CD103 modulation on cDCs in response to various stimuli (Nakano et al., 2013; Bernatchez et al., 2017; Brassard et al., 2019), the physiological function of this phenomenon on the host immune response remained undefined. We propose here that the absence of CD103 expression supports the intensity of the initial host innate immune response. Anecdotally, in a model of skin bacterial infection, cDC1s were crucial for neutrophils recruitment to the site of infection *via* the secretion of VEGF-α (Janela et al., 2019), which suggested that cDC1s influence neutrophil migration. CD103 signaling in cDC1s could be involved in promoting homeostasis by restraining the production of chemokines or pro-inflammatory cytokines. In contrast, during bacterial infections, the absence of CD103 signaling on cDC1s could increase their capacity to secrete neutrophils-attracting chemokines or induce neutrophil migration *via* an indirect effect on surrounding cells. All in all, our study suggests that the reduced CD103 expression on cDC1s may be a step in stimulating the innate immune response to Gram-negative bacteria.

The higher recruitment of lung neutrophils in *Cd103^-/-^* mice in response to *P. aeruginosa* was not caused by a difference in the number or proportions of cDC or pre-DC populations. However, a reduced IRF4 expression in bone marrow pre-DCs was observed in *Cd103^-/-^* naive mice, suggesting a lower propensity of cDC2 differentiation at steady state in these mice. As pre-DCs don’t express CD103, an indirect mechanism must be involved to explain this (Brassard et al., 2019). Also, we cannot exclude the possibility that, as WT and *Cd103^-/-^* strains were not co-housed in our experiments, differences in microbiota could be involved in impacting IRF4 expression.

Neutrophils are crucial to eradicate *P. aeruginosa* infection (Koh et al., 2009). The fact that the increased neutrophil count in *Cd103^-/-^* mice did not in turn change bacterial clearance indicates that the number of neutrophils in WT mice was sufficient to quickly clear bacteria. This is also supported by the fact that most of this mucoid strain of *P. aeruginosa* was cleared from the lung past 6 h (data not shown) in WT mice. Furthermore, several other studies observed that a higher number of lung neutrophils do not necessarily lead to a better *P. aeruginosa* clearance (Ballinger et al., 2006; Sen-Kilic et al., 2019). As CD103 expression could restrain chemokine production by cDC1s, mice in which cDC1s constitutively express CD103 would be a great tool to address the importance of cDC-specific CD103 expression on neutrophil recruitment and bacterial clearance with WT mice. Based on current knowledge, it is hard to determine whether the alterations of cDC populations induced by *P. aeruginosa* promote or impair host immune responses. First, the combination of the important lung recruitment of cDC2s, known to secrete large amount of MIP-2 and KC neutrophil-attractant chemokines, and the possible higher capacity of cDC1s to recruits neutrophils in absence of CD103 expression suggest a positive impact on immune response. On the other hand, current knowledge on adaptive immune responses against bacterial infection endorse the idea that cDC1s support the efficacy of the adaptive immune response compared to cDC2s. To date, no studies have established a specific role for cDC1s or cDC2s in innate or adaptive immune responses to *P. aeruginosa*, but this report takes a first important step towards better understanding the modulation of local cDC populations in the context of Gram-negative bacterial clearance.

An important conclusion rising from this data and our previously published research is that several factors such as the presence of LPS, TNF or *P. aeruginosa* can prevent the induction of CD103 expression on cDC1 (Brassard et al., 2019). Currently, in the lung, CD103 remains one of the main markers used to identify cDC1s (Ng et al., 2018; Shekhar et al., 2018; Monaghan et al., 2020). However, our results suggest that CD103 is not an ideal marker for this population in context of lung inflammation as its expression is highly modulated. Furthermore, in a few other particular immunological contexts, some lung CD11b^+^ DCs can also express CD103, which could lead to misinterpretations if used as a specific marker of cDC1s (Shane et al., 2018; Tweedle and Deepe, 2018). Finally, another disadvantage is that CD103 is not a marker of human lung cDC1s (Guilliams et al., 2014; Amon et al., 2020). Thus, the ideal marker to properly identify cDC1s should be constitutively expressed by cDC1s independently of their differentiation stage or activation status, and similarly expressed by human cDC1s.

In summary, we demonstrated that i.n. exposure to the Gram-negative bacteria *P. aeruginosa* alters the proportions of CD103^+^ cDC1 and CD11b^+^ cDC2 populations in favor of cDC2s, *via* the modulation of bone marrow cDC precursors and an impact on CD103 mRNA production by cDC1s. Furthermore, we demonstrated that the absence of CD103 expression favors the recruitment of lung neutrophils, suggesting that this phenomenon could be an important step in the early innate immune response to *P. aeruginosa*.

## Data Availability Statement

The raw data supporting the conclusions of this article will be made available by the authors, without undue reservation.

## Ethics Statement

The animal study was reviewed and approved by Comité de protection des animaux de l’Université Laval.

## Author Contributions

JB and M-RB conceived the study. JB, JR, M-JB, and EB performed experiments. JB and M-RB drafted and revised the paper. AML and M-JB critically revised the manuscript. MV and CD contributed to the final revision and supported microbiology experiments. M-RB supervised the study. All authors contributed to the article and approved the submitted version.

## Funding

This work was supported by the *Fonds sur les Maladies Respiratoires Bégin/Lavoie de l’Université Laval* and by the *Fondation de l’Institut Universitaire de Cardiologie et de Pneumologie de l’Université Laval.*

## Conflict of Interest

The authors declare that the research was conducted in the absence of any commercial or financial relationships that could be construed as a potential conflict of interest.

## Abbreviations

BAL, Bronchoalveolar lavages; BMDC, Bone marrow derived dendritic cell; CFU, Colony forming unit; cDC, Conventional dendritic cell; FLT3L, FMS-like tyrosine kinase 3 ligand; FLT3L-BMDC, BMDC differentiated with FLT3L; FMO, Fluorescence minus one; GMߛCSF, Granulocyte-macrophage colony-stimulating factor; GM-CSFR, GM-CSF receptor; GM-CSFRα, GM-CSFR subunit α; GM-CSFRβ, GM-CSFR subunit β; i.n., Intranasal; LPS, Lipopolysaccharide; MFI, Mean fluorescence intensity; PBS, Phosphate buffered saline; TSB, Tryptic soy broth; WT, wild-type.
