# Supplementary material for: Exposure to the Gram-Negative Bacteria Pseudomonas aeruginosa Influences the Lung Dendritic Cell Population Signature by Interfering With CD103 Expression
Source: Front Cell Infect Microbiol. 2021 Jul 6;11:617481. doi: 10.3389/fcimb.2021.617481 (PMC8291145; doi:10.3389/fcimb.2021.617481)
Supplement: Supplementary file 1 [file DataSheet_1.docx]

**Supplementary Figures**

**
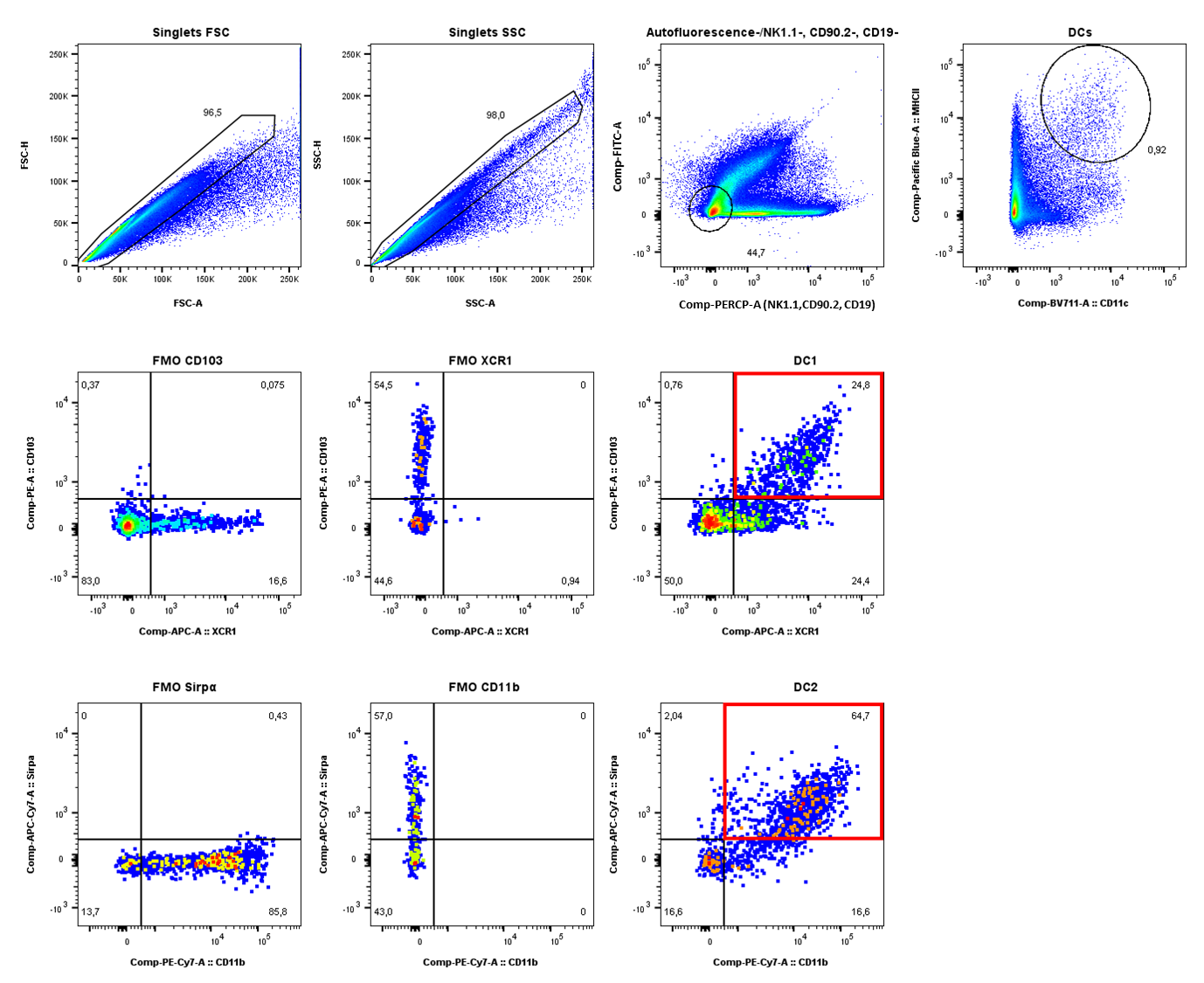
**

**Supplementary Figure 1: Flow cytometry-gating strategy for the identification of lung DC populations.** Single cells were identified by selecting the linear population in FSC-A/FSC-H panels, followed by the same process in SSC-A/SSC-H panel. Total DCs were identified as MHC II^Hi^ and CD11c^+^ from auto-fluorescence^-^, NK1.1^-^, CD90.2^-^, CD19^-^ cells. Cell positivity for CD103, XCR1, CD11b, Sirpα markers was determined using FMO control. CD103^+^ cDC1s were identified as CD103^+^XCR1^+^, while cDC2 were identified as CD11b^+^Sirpα^+^ from the total DC population.

**
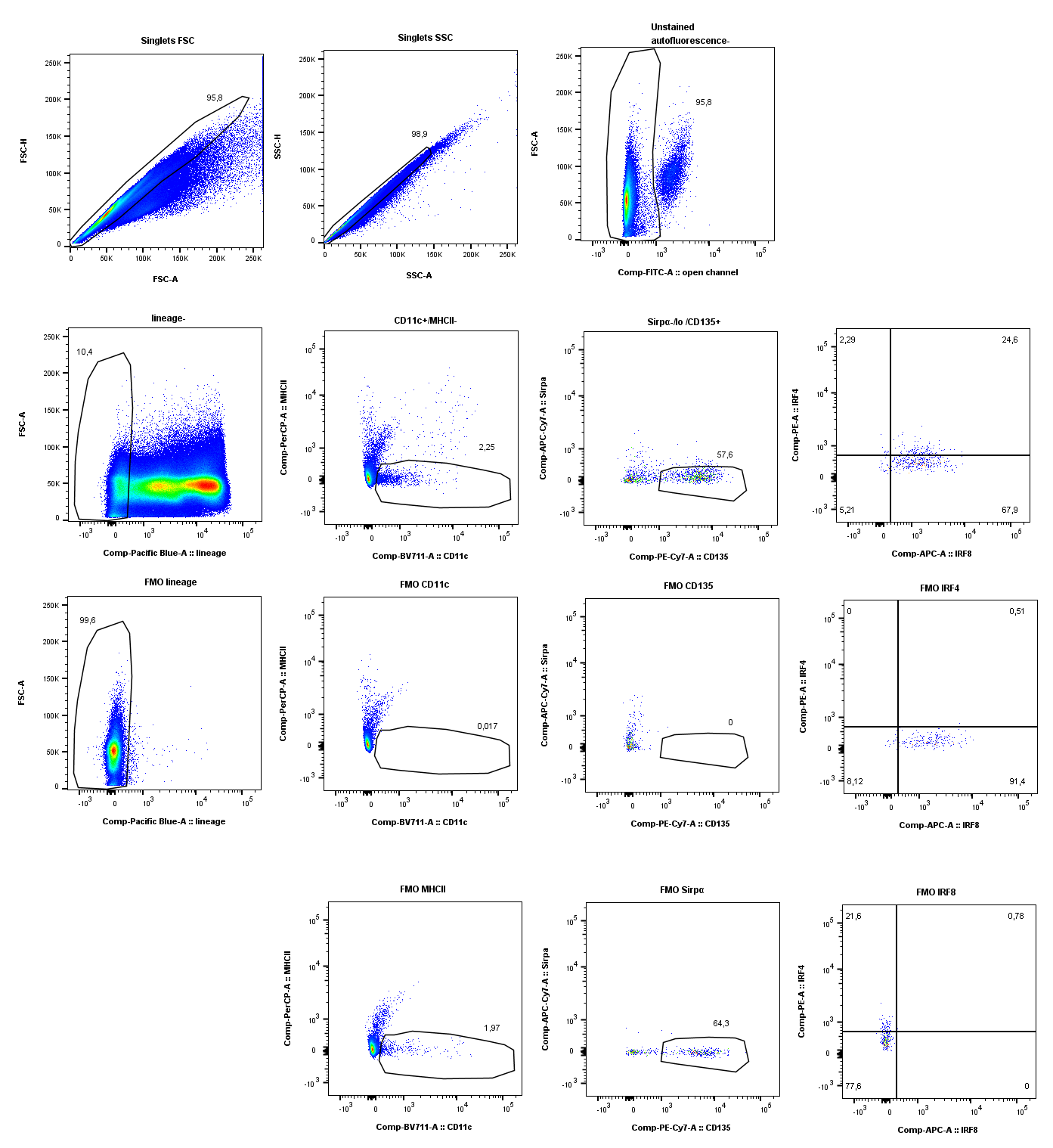
Supplementary Figure 2: Flow cytometry-gating strategy for the identification of bone marrow pre-DCs.** Single cells were identified by selecting linear population in FSC-A/FSC-H panel followed by the same process in SSC-A/SSC-H panel. Total pre-DCs were identified as auto-fluoresence^-^, lineage^-^, MHC II^-^, CD11c^+^, Sirpα^-/lo^ and CD135(FLT3)^+^. IRF8 and IRF4 expression was analysed in total pre-DCs. Positivity for all markers was determined using FMO controls.

**
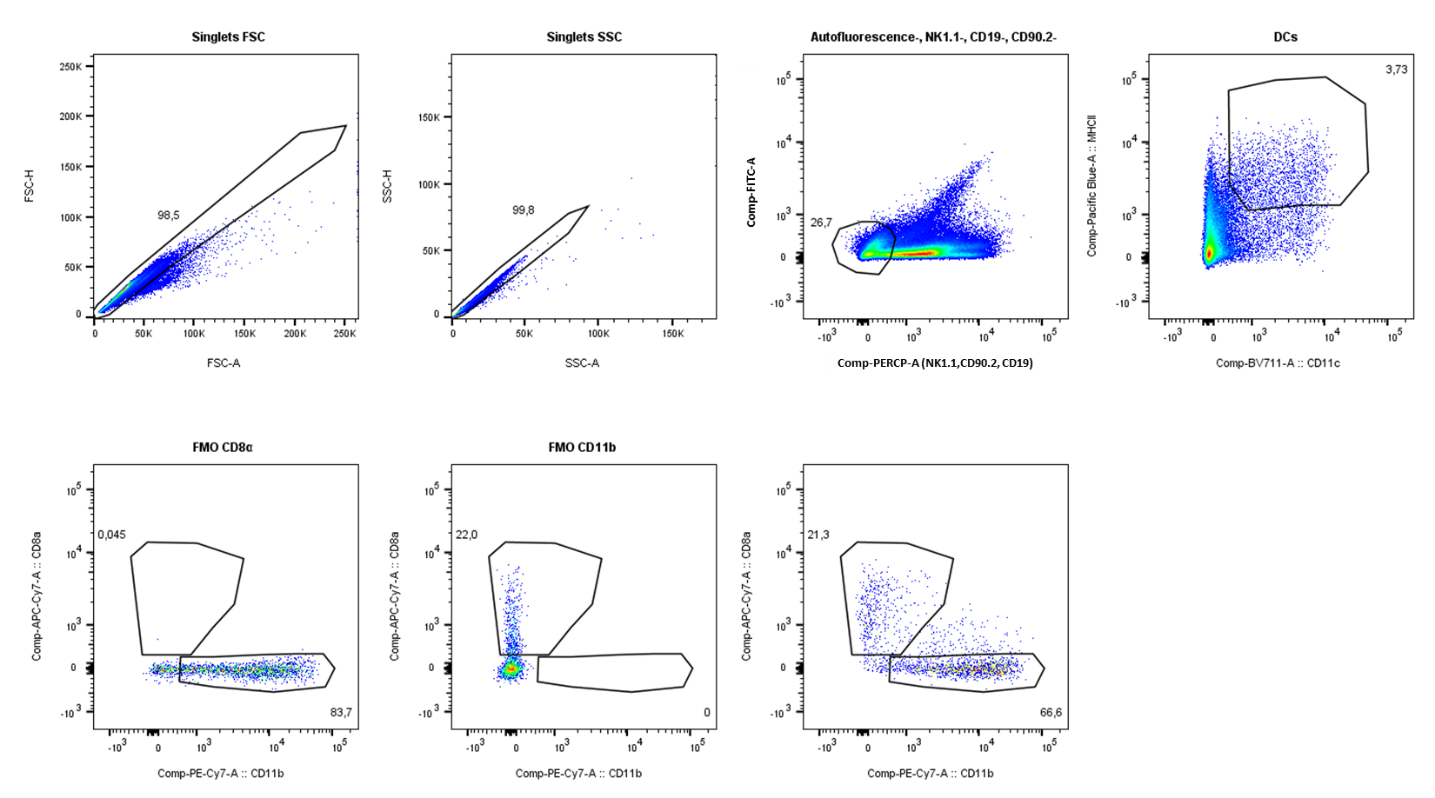
Supplementary Figure 3: Flow cytometry-gating strategy for the identification of splenic DCs.** Single cells were identified by selecting linear populations in the FSC-A/FSC-H panel followed by the same process in the SSC-A/SSC-H panel. Total DCs were identified as MHC II^Hi^ and CD11c^+^ from auto-fluorescence^-^, NK1.1^-^, CD90.2^-^, CD19^-^ cells. Cell positivity for CD8α and CD11b was determined using FMO controls. cDC1s were identified as CD8α^+^CD11b^-^, while cDC2s were identified as CD11b^+^CD8α^-^ .

**
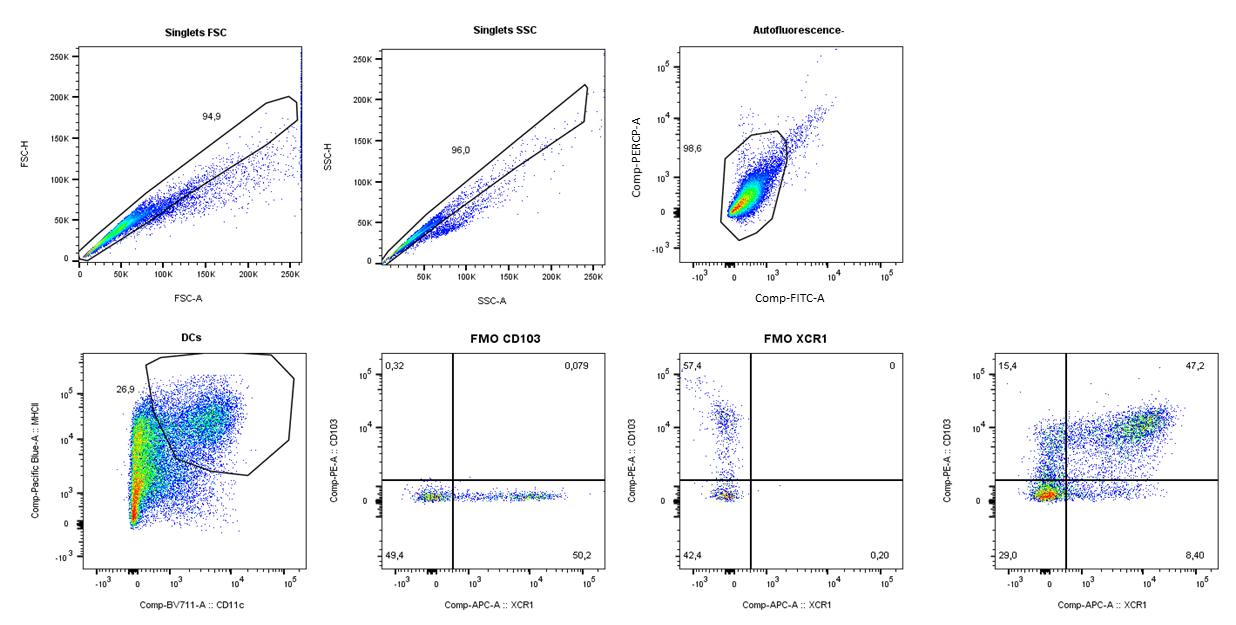
Supplementary Figure 4:** **Flow cytometry-gating strategy for the analysis of CD103 expression on XCR1^+^ FLT3L-BMDCs.** Single cells were identified by selecting linear population in the FSC-A/FSC-H panel followed by the same process in the SSC-A/SSC-H panel. Total DCs were identified as MHC II^Hi^ and CD11c^+^ from auto-fluorescence^-^ cells. Cell positivity for CD103 and XCR1 markers was determined using FMO controls.
